# Squamous Cell Carcinoma of the External Auditory Canal: A Case Report

**DOI:** 10.1155/2011/615210

**Published:** 2011-10-19

**Authors:** Harry Boamah, Glenn Knight, Joseph Taylor, Kevin Palka, Billy Ballard

**Affiliations:** ^1^Department of Medical Student, Meharry Medical College, Nashville, TN 37208, USA; ^2^Department of Radiology, Meharry Medical College, Nashville, TN 37208, USA; ^3^Department of Surgery, Meharry Medical College, Nashville, TN 37208, USA; ^4^Department of Internal Medicine, Meharry Medical College, Nashville, TN 37208, USA; ^5^Department of Medicine, Vanderbilt University Medical Center, Nashville, TN 37232, USA; ^6^Department of Pathology, Meharry Medical College, Nashville, TN 37208, USA

## Abstract

Squamous cell carcinoma of the temporal bone and external auditory canal is a rare tumor with a reported incidence of between 1 to 6 cases per million population per year. Because squamous cell carcinoma of the temporal bone and auditory canal is so rare, developing an adequate tumor staging system and treatment has been difficult. We present a case of squamous cell carcinoma of the external auditory canal in 65-year-old Hispanic female who presented with a 6-month history of right ear pain, 3-month history of serosanguineous right ear drainage, and symptoms of facial paralysis. Due to the extensive spread of her tumor into the middle ear at the time of diagnosis, her tumor was deemed unresectable and she received palliative chemotherapy and radiation therapy and was sent to Alice Hospice and died several weeks later.

## 1. Introduction

Carcinoma arising in the external auditory canal and temporal canal is rare and comprises <0.2% of head and neck neoplasms with an age adjusted incidence of 1/1,000,000 per year in women and 0.8/1,000,000 in men reported in England, Wales, and the USA. Squamous cell carcinoma is the most common pathology, which accounts for 0–90 percent of neoplasms of the temporal bone [1]. This is an aggressive disease with the prognosis dependent on the stage of disease and the primary treatment. Other primary histologic types of neoplasms arising in the external auditory canal and temporal bone include adenocarcinoma, adenoid cystic carcinoma, mucoepidermoid carcinoma, basal cell carcinoma, ceruminous carcinoma, and rhabdomyosarcoma. Chronic otorrhea and inflammatory or cholesteatoma within the external auditory canal and middle ear are associated risk factors. The following is a case report of one such patient who presented to this institution with squamous cell carcinoma of the external auditory canal. 

## 2. Case Report

A 65-year-old Hispanic female presented to emergency department (ED) with a 6-month history of right ear pain that did not respond to ciprodex and keflex. The patient described initial drainage of clear fluids which became serosanguineous over the last 3 months. The patient denied any history of smoking or alcohol abuse.

In the ED, otoscopic examination of the right ear showed that the external auditory canal was edematous with clear discharge and bulging of the tympanic membrane. The patient was found to have decreased sensation over the right side of the face in the second and third trigeminal region but normal sensation over the right forehead. She had right eye ptosis and paralysis. The patient was unable to protrude her tongue, and there appeared to be a facial droop over the right side.

**Figure 1:**
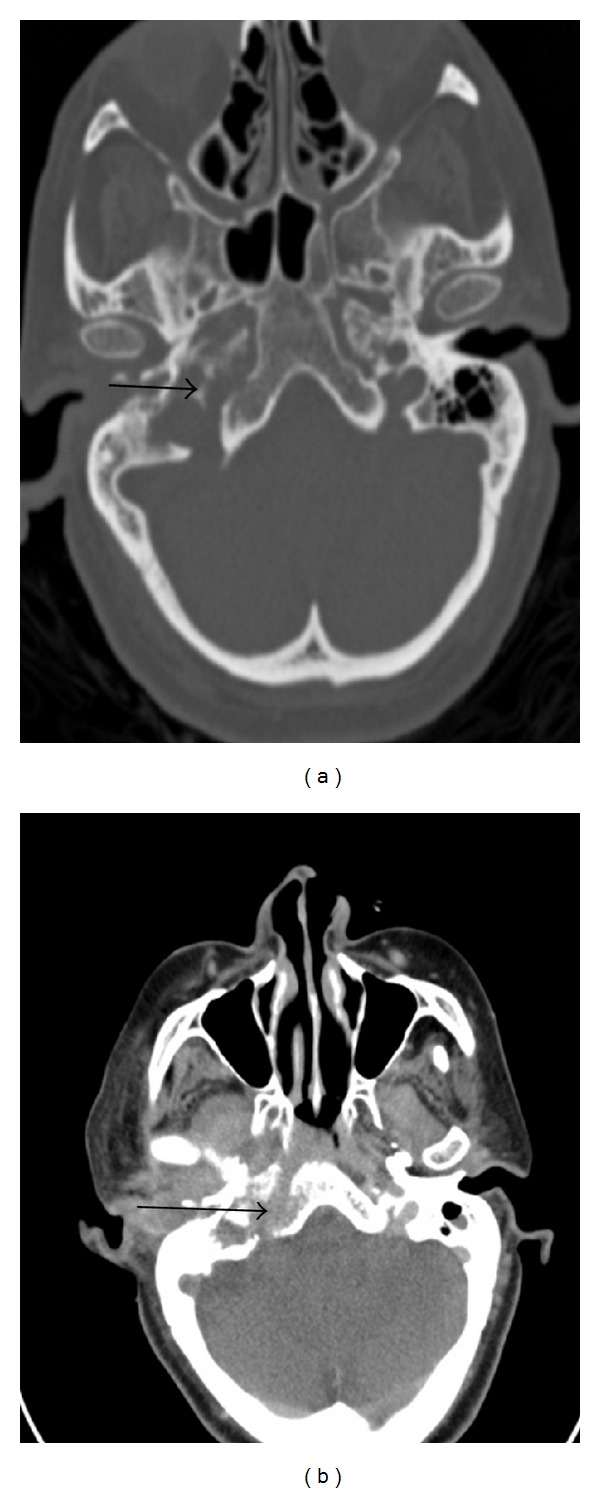
Right inner ear and temporal bone computed tomography scan on first admission (a) and on readmission (b) showing opacification of the right external auditory canal, right tympanic, and epitympanic spaces, as well as the right mastoid antrum. There is also bony destruction of the anterior wall of the right external auditory canal, scutum and wall of the right carotid canal, foramen lacerum, and semicircular canals. The arrows show the extent of tumor spread into the middle ear.

 A CT scan of her right inner ear and temporal bone showed opacification of the right external auditory canal and bony destructive changes of the anterior wall of the right external auditory canal, right tympanic and epitympanic spaces, and mastoid antrum. The CT also showed destructive changes of the anterior wall of the right external auditory canal, scutum wall of the right carotid canal and foramen lacerum, and wall of the right semicircular canal (Figure 1).

**Figure 2:**
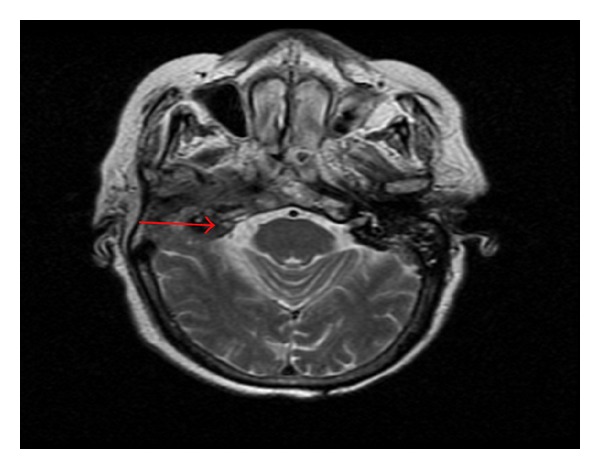
Neck soft tissue Magnetic resonance imaging showing heterogeneous signal abnormality and enhancement centered about the right external auditory canal and middle ear region. The arrow depicts spread of the tumor into the middle ear.

A magnetic resonance imaging scan of the neck revealed extensive heterogeneous signal abnormality and enhancement centered about the right external auditory canal and middle ear region (Figure 2). Similar abnormality and enhancement were noted in the right temporal bone extending into the carotid and parapharyngeal spaces. There was also an equivocal 9 × 7 mm fluid collection in the carotid space and associated dural enhancement in the sigmoid sinus. The patient was thought to have a severe otitis media and was treated with antibiotics and was to follow up with the ear, nose, and throat clinic as an outpatient. 

**Figure 3:**
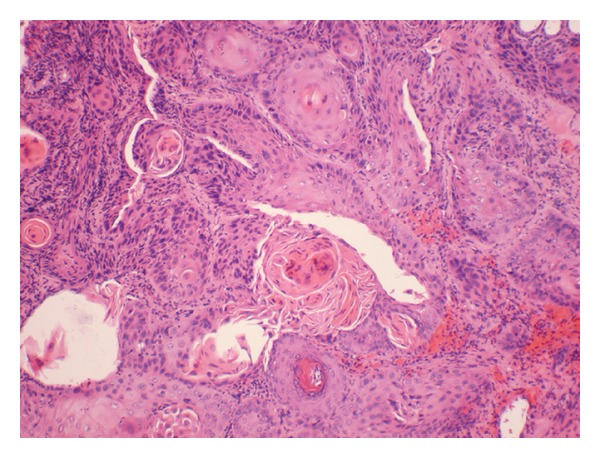
Showing well-differentiated squamous cell carcinoma, characterized by sheets of cells with large cells with pleomorphic, hyperchromatic nuclei and individual cell keratinization, and keratin pearl formation.

However, the patient was readmitted again three weeks later with symptoms of nausea and vomiting and worsening of the right ear pain. On readmission, the patient was found to be hyponatremic with altered mental status. The lesion of the right external auditory canal was biopsied. The pathology report showed a well-differentiated squamous cell carcinoma (Figure 3).

The patient underwent a positive electronic transmission scan of the full body which showed that her tumor was confined to the right external and middle ear with no metastasis. 

The patient had stage T4NO disease based on radiological CT imaging of the right ear which showed that the tumor has penetrated through the right external auditory canal into the scutum and walls of the right carotid and foramen lacerum. There was also evidence on the CT and MRI showing local invasion of the facial nerve in the middle ear (Figure 2); this was thought to be the cause of her facial paralysis.

The patient was evaluated by head and neck surgery, and her tumor was found to be unresectable due to the extensive local invasion. The patient received induction chemotherapy consisting of carboplatin, paclitaxel, and cetuximab for 2 cycles before getting palliative short-course radiation therapy. The patient was referred to alive hospice for palliative care and died several weeks later.

## 3. Discussion

Squamous cell carcinoma of the temporal bone and external auditory canal is a rare tumor with a reported incidence of between 1 to 6 cases per million population per year [1]. It accounts for less than 0.2% of all tumors of the head and neck [2], but is the most common neoplasm in the external auditory canal [1]. In general, tobacco and alcohol use are the two most important risk factor associated with squamous cell cancers of the head and neck. Squamous cell carcinoma of the temporal bone and external auditory canal is often associated with chronic otitis media and exposure to radiation therapy [1]. The diagnosis of squamous cell carcinoma of the temporal bone and external auditory canal is based on histological examination of tissue of the tumor from the ear.

 Because squamous cell carcinoma of the temporal bone and auditory canal is so rare, developing an adequate tumor staging system and treatment has been difficult. To date, there is no universally accepted staging system for cancers of the temporal bone [3]. The University of Pittsburgh staging system for primary squamous cell cancer of the external auditory canal was proposed by Arriaga et al. in 1990 to attempt to classify the disease prior to treatment [4]. Although it has its limitations for determining soft tissue extension of the tumor, the development of a TNM staging system using preoperative high-resolution CT scans of the temporal bone has been confirmed by other studies to accurately reflect the extend of the disease and planning of tumor resection [5]. Gillespie et al. in 2001 published a retrospective chart review of 15 patient treated for squamous cell carcinoma and reported that the University of Pittsburgh staging system correlated with patient outcome and was more sensitive than preoperative radiological staging [6]. 

The staging of squamous cell cancer of the external auditory canal is based on the extent of tumor spread in the ear and temporal bone. Based on the Pittsburgh tumor staging system for squamous cell carcinoma of the temporal bone, T4 tumors are those that have eroded the cochlear, petrous apex, medial wall of the middles ear with extension of the carotid canal, jugular foramen or dura, or tumors with extensive (>0.5 cm) soft tissue involvement [6]. Recently, Moody et al. proposed that the classification of T4 tumors includes those involving the facial nerve. The modified Pittsburgh staging of squamous cell cancer of the temporal bone classifies patients who present with facial paralysis as T4 tumors [3]. 

The treatment of squamous cell cancer of the external auditory canal depends on the staging of the tumor which includes lymph nodes metastasis and facial nerve involvement. The preferred treatment often consists of a combination of en bloc surgical resection of the primary tumor with tumor-free surgical margins and postoperative chemotherapy and radiotherapy. The surgery that is often performed is the lateral temporal bone resection (LTBR) or a subtotal temporal bone resection (STBR) [7]. Poor prognostic factors include the extent of the disease at presentation, positive margin, dural and cranial nerve involvement, and facial nerve paralysis. The overall 5-year survival rate of individuals with squamous cell cancer of the temporal bone ranges between 40% and 70% but can reach 20% in advance stage diseases [2].

## 4. Conclusion

Squamous cell carcinoma of the external ear canal and temporal canal is very rare, and their treatment depends on the stage of the tumor. Early detection of the tumor before extensive spread into the middle ear allows for better treatment and better prognosis. 

At presentation, this patient had a T4NO squamous cell cancer of the external auditory canal with no evidence of metastasis. T4 tumors squamous cell cancers of the external auditory canal have a very poor prognosis; the two-year survival of T4 squamous cell cancers of the external auditory canal is reported to be between 0–7% [3]. Due to the extensive spread of the tumor into the middle ear at the time of diagnosis, this patient's tumor was deemed unresectable and she received palliative chemotherapy and radiation therapy and was sent to Alice Hospice for palliative care.
